# A Protocol for the In Vitro Culturing of Vascularized Pancreatic Islet Organoids

**DOI:** 10.3390/bioengineering12111222

**Published:** 2025-11-09

**Authors:** Pengkun Song, Yue Wang, Junya Peng, Lei Liu, Lei Du

**Affiliations:** 1 State Key Laboratory of Organ Regeneration and Reconstruction, Institute of Zoology, Chinese Academy of Sciences, Beijing 100101, China; songpengkun21@ioz.ac.cn (P.S.);; 2 University of Chinese Academy of Sciences, Beijing 100049, China; 3 State Key Laboratory of Complex, Severe, and Rare Diseases, Peking Union Medical College Hospital, Beijing 100730, China; 4 Beijing Institute for Stem Cell and Regenerative Medicine, Beijing 100101, China

**Keywords:** islet organoids, HUVEC, vascularization, insulin secretion

## Abstract

This study presents a protocol for co-culturing pancreatic islet cell clusters derived from pancreatic tissue with human umbilical vein endothelial cells (HUVECs) on Matrigel using a specialized culture medium to form vascularized pancreatic islet organoids. We established a novel culture system for vascularized pancreatic islet organoids and compared the survival and insulin secretion capabilities of pancreatic islet cells in the presence and absence of glucose stimulation. Our results indicate that matrix adhesive materials can effectively facilitate the self-assembly of the vascularized endothelial cell–pancreatic islet organoids complex. Vascularized HUVEC prolongs the survival of pancreatic islet organoids in vitro. Moreover, the interaction between vascularized HUVEC and pancreatic islets significantly enhances the insulin secretion ability in response to glucose stimulation. This study reports a protocol for the long-term in vitro culture of pancreatic islet organoids, offering methods for the vascularization of pancreatic islet organoids on Matrigel. These data contribute to the understanding of how vascularization impacts the fate and function of pancreatic islet organoids, although the specific mechanism still requires further clarification.

## 1. Introduction

In native islets, hormone-producing endocrine cells are encompassed by a vascular network composed of endothelial cells (ECs), stromal cells, neurons, and immune cells [1,2,3]. The vascular network that surrounds and penetrates each islet facilitates β-cell glucose sensing and the release of insulin (INS) into circulation [4]. A prevalent approach for attaining organoid vascularization is to cultivate human umbilical vein endothelial cells (HUVECs) in conjunction with other constituent cells [5,6]. For instance, researchers successfully accomplished the guided differentiation process of human embryonic stem cells (hESCs) and co-cultured them with HUVECs to develop a vascularized neural tissue model [7,8,9].

The extracellular matrix (ECM) components and soluble factors secreted by ECs create a local microenvironment within the islet that furnishes essential signals for β cell function. The direct interaction of β cells with the basement membrane ECM stimulates integrin-β1 signaling, which serves to regulate multiple facets of β cell function, including INS production, Ca^2+^ influx, and INS exocytosis [10,11]. Furthermore, the soluble factors derived from ECs provide important paracrine signals for β cell function. When isolating islet from the pancreas and during in vitro culture, the islet vasculature is rapidly lost, leading to impaired β cell function in cultured islets [5,12,13,14]. Despite the fact that the vasculature is of crucial importance for islet function, no in vitro model can replicate the three-dimensional vascular network of the native islet niche at present.

The emergence of islet organoids via organoid technology represents a timely development that has garnered considerable interest and is recognized as a valuable asset for advancing diabetes research. Pancreatic islet organoids derived from adult stem cells or tissue progenitor cells are founded on the current understanding of the development mechanism of the pancreas. During the development process, stem cells can be induced to differentiate into three-dimensional cell cultures with a structure and function similar to those of natural islets by programmatically activating or inhibiting certain signaling pathways and adding inducing factors in vitro [15,16]. Moreover, numerous research experiments conducted both domestically and internationally have demonstrated that vascular endothelial cells exert a regulatory effect on the physiological state of cultured pancreatic islets in vitro [17]. Due to their capability to simulate the physiological activities of the pancreas to a certain extent in vitro, islet organoids can reflect the tissue structure of pancreas, perform the physiological functions of secreting multiple hormones, and regulate blood glucose levels [18]. Here we isolated pancreatic islets, and subsequently established a vascularized islet organoid model. In this model, human umbilical vein endothelial cells (HUVECs) spontaneously formed an organized vascular network surrounding the β cells. Notably, we were able to maintain these vascularized pancreatic organoids in vitro for an extended period. It was found that this vascular network enhances the function of β cells. Matrigel materials effectively promotes the construction of vascularized pancreatic islet organoids. Cell survival experiments have demonstrated that HUVECs can prolong the survival of pancreatic islet organoids during in vitro culture. Co-culture with HUVECs significantly enhances the insulin secretion capacity of islet organoids in response to glucose stimulation.

## 2. Materials and Methods

### 2.1. Tissues and Cells

Pancreatic tissue samples were collected from Peking Union Medical College Hospital with informed consent from the donors. Vascular endothelial cells (HUVEC, HMEC, and HPAEC) were obtained from commercial sources (Fuheng Biotechnology, Shanghai, China) and cultured in Complete Endothelial Cell Medium (ScienCell, 1001, Carlsbad, CA, USA) at 37 °C in a humidified incubator supplemented with 5% CO_2_. All experiments were approved by the Biomedical Research Ethics Committee of the Institute of Zoology, Chinese Academy of Sciences.

### 2.2. Acquisition of Pancreatic Islet Samples

Pancreatic tissue was first rinsed with sterile culture medium to remove blood contaminants, then mechanically minced into approximately 1–2 mm^3^ fragments. Tissue digestion was performed in a freshly prepared digestive cocktail containing: 10mL DMEM (Gibco, Grand Island, NY, USA), 1mg/mL Collagenase P (Roche, 11249002001, Basel, Switzerland), 0.1mg/mL DNase I (Sigma-Aldrich, DN25-1G, St. Louis, MO, USA). The tissue suspension was incubated in a 37 °C water bath with continuous gentle agitation every 2 min. Digestion progress was monitored every 5 min, and terminated when >90% of pancreatic tissue was dissociated while preserving islet integrity (verified by light microscopy). The reaction mixture was allowed to settle by gravity for 2 min. The supernatant was carefully collected and transferred to a 15 mL centrifuge tube, then subjected to low-speed centrifugation (300× *g* for 1 min). The supernatant was centrifuged at 500× *g* for 3 min to pellet the islet-enriched fraction. The resulting islet cells were cultured in a specialized islet culture system (composition detailed in Table 1) to facilitate pancreatic organoid formation.

### 2.3. Immunofluorescent Staining

Cells were washed three times with phosphate-buffered saline (PBS), followed by fixation in 4% paraformaldehyde (PFA) for 1 h at 37 °C. After fixation, cells were permeabilized with 0.5% Triton X-100 for 20 min. For immunofluorescence staining, cells were incubated overnight at 4 °C with the following primary antibodies: anti-Insulin (1:200, Cell Signaling Technology, 8138S, Danvers, MA, USA), anti-Glucagon (1:200, Cell Signaling Technology, 2760S), and anti-CD31 (1:200, Biolegend, 303104, San Diego, CA, USA). After washing, cells were incubated with fluorescence-conjugated secondary antibodies for 2 h at room temperature in the dark. Subsequently, cells were counterstained with 4′,6-diamidino-2-phenylindole (DAPI, 1 μg/mL) for 5 min, mounted onto glass slides using an anti-fade mounting medium, and imaged using a confocal microscope (Zeiss LSM880, Oberkochen, Germany).

### 2.4. Calcein and Propidium Iodide (PI) Staining

A total of 30 manually selected pancreatic islets were seeded per well, with experimental groups divided into: (1) a control group (islets cultured in isolation) and (2) an experimental group (islet clusters co-cultured with vascular endothelial cells). From day 2 post-seeding, islet viability was dynamically assessed on days 2, 5, and 10 using a dual fluorescent staining assay. Live and dead cells were simultaneously visualized by staining with Calcein-AM (1:1000, Beyotime, Y27151, Shanghai, China) and Propidium Iodide (PI, 1:500, Beyotime, ST512). Stained islets were immediately examined under an inverted fluorescence microscope, and cell viability was quantified as the percentage of Calcein-AM-positive (live) cells relative to the total cell population (live + dead) through manual counting.

### 2.5. Glucose-Stimulated Insulin Secretion Test (GSIS)

Equally sized islets (n = 30 per group; ~400 μm diameter) from both experimental and control groups were subjected to a standardized GSIS protocol. Briefly, islets were washed three times with glucose-free Krebs-Ringer Bicarbonate Buffer (KRBB, Coolaber, SL6561, Beijing, China) and pre-incubated in low glucose (2 mM) KRBB for 2 h to deplete residual insulin. After a brief rinse in glucose-free KRBB, islets were further incubated in low-glucose (2 mM) KRBB for 30 min to stabilize basal secretion. Islets were then washed twice with glucose-free KRBB and incubated in high-glucose (20 mM) KRBB for 30 min to stimulate insulin release. The supernatant was carefully collected for insulin quantification via ELISA (Beyotime, PI608). Data validity was ensured by applying a 20% variability threshold across technical replicates. Only results where inter-assay differences remained within this range were considered reliable, with the final insulin concentration reported as the mean of qualified measurements.

### 2.6. Reverse Transcription Quantitative PCR Analysis

Total RNA was isolated using TRIzol™ reagent (Invitrogen, 15596026, Waltham, MA, USA) following the manufacturer’s protocol. RNA purity and concentration were assessed by spectrophotometry. First-strand cDNA was synthesized from 1 μg of total RNA using a reverse transcription kit (YEASEN, 11151ES60, Gaithersburg, MD, USA), strictly adhering to the supplier’s instructions. Gene expression levels were quantified using SYBR^®®^ Green-based qPCR (YEASEN, 11184ES08) on a real-time PCR system. Gene expression was normalized to β-actin using the 2^−ΔΔCt^ method.

Primer sequences were as follows: INS (insulin): Sense: 5′-ACGAGGCTTCTTCTACACACCC-3′, antisense: 5′-TCCACAATGCCAGCTTCTGCA-3′. Glucagon primer, Sense: 5′-CGTTCCCTTCAAGACACAGAGG-3′, antisense: 5′-ACGCCTGGAGTCCAGATACTTG-3′. β-actin primer, Sense:5′-CACCATTGGCAATGAGCGGTTC-3′, antisense: 5′-AGGTCTTTGCGGATGTCCACGT-3′. The thermal cycling conditions were: 95 °C for 10 min (initial denaturation), followed by 40 cycles of 95 °C for 15 s and 60 °C for 1 min (annealing/extension).

### 2.7. Immunohistochemistry (IHC) Analysis

Formalin-fixed, paraffin-embedded pancreatic tissues and islet organoids were cut into 5 μm-thick sections. Sections were dewaxed, rehydrated, and subjected to antigen retrieval using Tris-EDTA buffer (pH 8). After blocking with 5% milk for 30 min, sections were incubated overnight at 4 °C with the following primary antibodies: anti-Insulin (1:1000, Cell Signaling Technology, 8138S), anti-Glucagon (1:1000, Cell Signaling Technology, 2760S), anti-SST (1:1000, SIGMA, HPA019472), anti-PPY (1:1000, SIGMA, SAB2500747), anti-C-peptide (1:1000, Cell Signaling Technology, 4593S). After PBS washes (3 × 5 min), sections were incubated with horseradish peroxidase (HRP)-conjugated secondary antibodies (ZSGB-Bio, PV-9000, Beijing, China) for 30 min at room temperature. 3,3′-Diaminobenzidine (DAB, ZSGB-Bio, ZLI-9019) was applied according to the manufacturer’s instructions to develop brown precipitates. Sections were counterstained with hematoxylin (ZSGB-Bio, ZLI-9610) for 1–2 min, and dehydrated through graded ethanol and xylene. Stained sections were coverslipped using a non-aqueous mounting medium, and examined under a light microscope.

### 2.8. Statistical Analysis

Statistical analysis was performed using Student’s *t*-test. A *p*-value of less than 0.05 was deemed statistically significant, with * *p* < 0.05, ** *p* < 0.01, and *** *p* < 0.001 denoting significance levels compared to controls. All statistical calculations were performed using GraphPad Prism 9.5 software.

## 3. Results

### 3.1. Culture of Pancreatic Islet Cells

**Figure 1:**
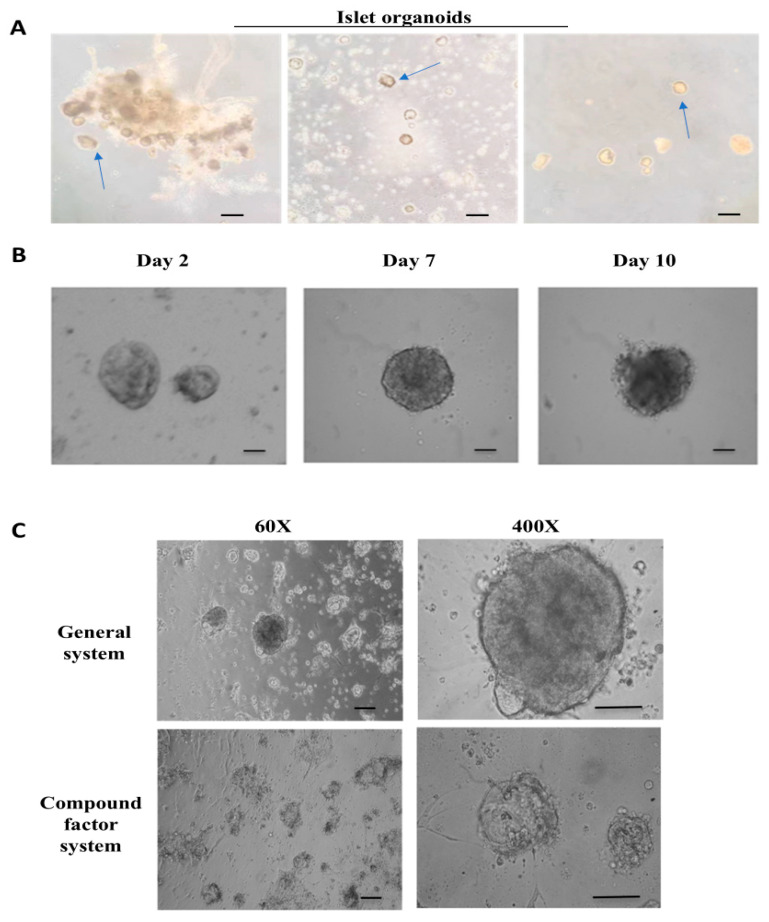
Isolation and culture of islet cells. (**A**) The process of in vitro isolation of pancreatic islet cells. From left to right, it involves the encapsulation of pancreatic islet cell clusters by exocrine cells, and the subsequent mixing of pancreatic islet cell clusters with exocrine cells to form individual pancreatic islet cell clusters. The arrow in the figure indicates the pancreatic islet cluster. Scale bar, 50 μm. (**B**) The cell morphology of pancreatic islet cells cultured in vitro on the 2nd, 7th, and 10th days (from left to right). Scale bar, 20 μm. (**C**) The cell morphology of pancreatic islet organoids cultured under traditional and composite systems on the 5th day. Scale bar, 50 μm.

Normal pancreatic tissue samples were collected from clinical surgery specimens. The isolated pancreatic islet cell clusters were cultured in three-dimensions on Matrigel, supplemented with essential growth factors, forming pancreatic islet organoids exhibiting robust viability and normal physiological functions. We observed that the contaminating exocrine cells and tissue fragments during the separation procedure significantly impaired the growth of pancreatic islet clusters on Matrigel. Specifically, the residual tissue fragments, certain connective tissues, and adipose tissues generated during the cutting and grinding stages will adhere to and enwrap the pancreatic islet clusters, causing a rapid decline in the state of the islets during in vitro culture and their fundamental demise within a week. To address this, we further optimized the separation method by adopting multiple centrifugation (600 rpm → 1000 rpm) and filtration steps, which can effectively isolate exocrine cells from pancreatic tissue and yield pancreatic islet cell clusters of higher purity (Figure 1A). Compared to conventionally cultured controls, the pancreatic islet cell clusters maintained in the composite factor culture system showed enhanced survival capacity and physiological activity (Figure 1B).

Simultaneously, we established a long-term in vitro culture system for islet organoids, maintaining functional viability for approximately 14 days. Culture conditions were controlled at 37 °C with 5% CO_2_ supplementation. The optimized medium contained specific growth factors essential for islet cell viability, including: advanced DMEM/F12 as the basal nutrient source for cellular metabolic processes, FGF2 to promote cell proliferation and tissue neovascularization, EGF as a mitogenic factor, VEGF for angiogenesis induction, and ITS-X to regulate growth signaling and nutrient utilization (Table 1; Figure 1C).

Subsequently, we examined the cell morphology and physiological activity of pancreatic islet organoids cultured in this system on days 2, 7, and 10. The findings indicated that on the 7th day, the pancreatic islets maintained a favorable cell morphology, and the surface of the pancreatic islet clusters remained intact. Starting from the 10th day, the surface structure of the pancreatic islet clusters sustained damage, and the surface began to rupture, the pancreatic islet cells underwent varying degrees of death (Figure 1B). Based on these findings, islet samples cultured for 2, 7, and 10 days were selected as time points for subsequent biochemical assays.

### 3.2. Specific Antigen Detection of Pancreatic Islet Cells

**Figure 2:**
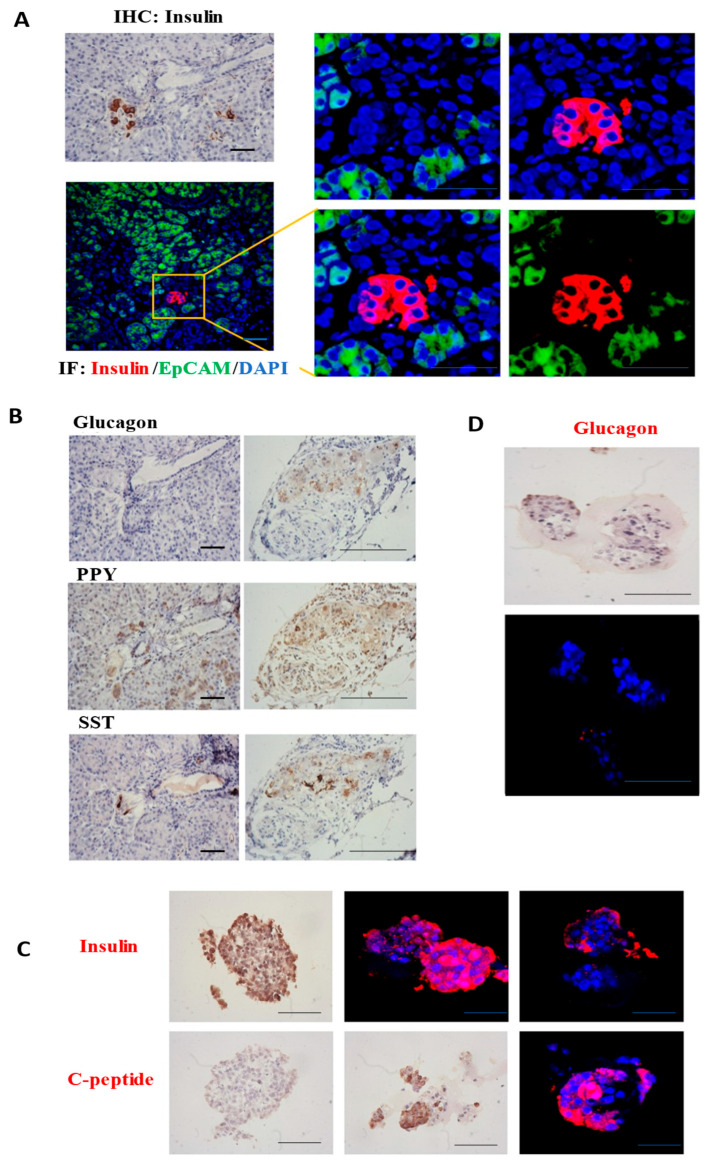
Specific antigen detection of pancreatic islet cells in tissues. (**A**) Immunohistochemistry (IHC) was employed to detect the insulin protein specifically secreted by β cells in pancreatic islets within pancreatic tissue. Immunofluorescence (IF) detection and identification of pancreatic islets β cells were carried out, with a bar scale of 50 μm. (**B**) IHC staining of glucagon, PPY, SST to detect α cells, PP cells, and δ cells within pancreatic tissue, respectively. (**C**) IHC and IF staining of insulin and c-peptide to detect β cells within pancreatic islet organoids. (**D**) Detection of glucagon antigen in α cells within pancreatic organoid islets. Scale bar, 20 μm.

In the culture system of pancreatic islet cells, we conducted immunohistochemical (IHC) detection of specific antigens in the islet cell clusters within the obtained pancreatic tissue. The pancreatic tissue underwent fixation, dehydration, embedding, and slicing procedures to acquire tissue samples for the detection of specific antigens. Specific antibodies against insulin, C-peptide, glucagon, PPY, and SST were employed for this detection. The results indicated the presence of numerous islet cell clusters in the pancreatic tissue, suitable for subsequent isolation of islet-like organoids (Figure 2A). Clustered human islet cells were identified within the pancreatic tissue. The four types of islet cells were aggregated together and enveloped by a layer of membrane. The islet cell clusters were dispersed throughout the pancreatic tissue composed of exocrine cells, accounting for ~1% of the total pancreas and expressing corresponding antigens (Figure 2B). This detection provides a theoretical basis and experimental foundation for subsequent sampling and in vitro culture of pancreatic islets.

After determining the location and approximate proportion of the islet clusters within the pancreatic tissue, the isolated islet samples were cultured on Matrigel. IHC analysis of islet organoids confirmed expression of glucagon (α-cells), insulin (β-cells), and C-peptide. Our findings indicate that islet organoids derived from isolated clusters exhibit physiological states consistent with native human islets and express specific antigens. The islet organoids express specific antigens of the islets. However, the cell viability of the islet α cells is relatively low, whereas the insulin secretion ability of the islet β cells is robust. During in vitro culturing, the glucagon secretion capacity decreases significantly (Figure 2C,D).

### 3.3. Culture and Identification of Endothelial Cells

**Figure 3:**
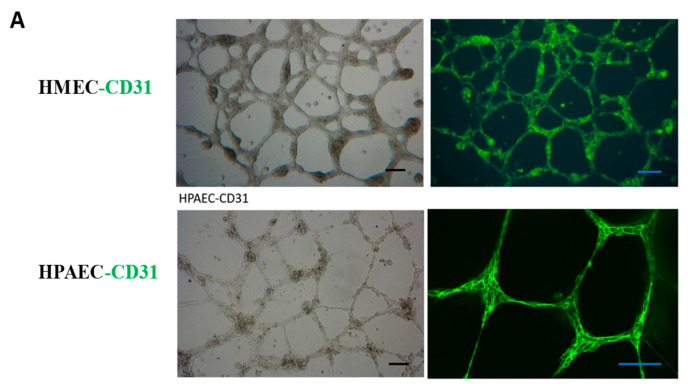

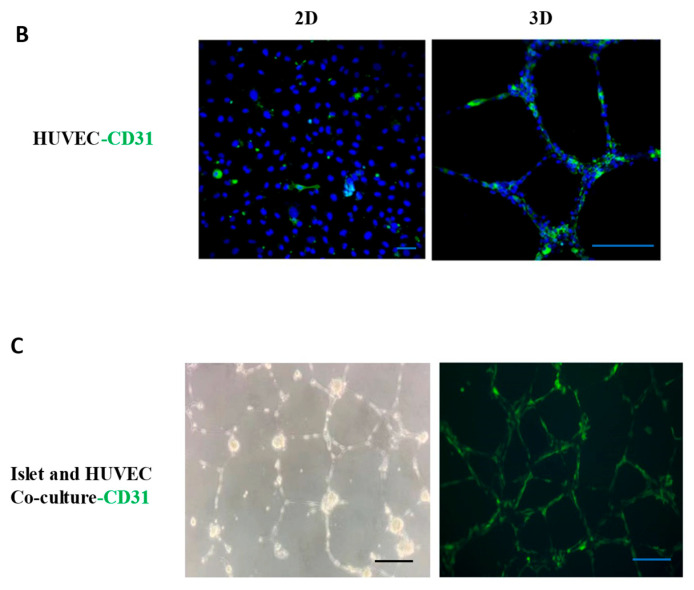
Culture and identification of endothelial cells. (**A**) Identification of endothelial marker CD31 in HMEC and HPAEC. Scale bar, 50 μm. (**B**) HUVEC were cultured in 2D and 3D environment, and the endothelial marker CD31 was detected by IF. Nuclei were counterstained with DAPI (blue). Scale bar, 50 μm. (**C**) A network structure formed by the co-culture of pancreatic islets and HUVEC was observed under light microscopy, with endothelial cells enveloping pancreatic islet clusters. After the co-culture of pancreatic islets and endothelial cells, the expression of HUVEC marker molecule CD31 was detected by IF.

In order to more effectively cultivate islet organoids and facilitate subsequent endothelial cell detection, we cultured and identified three types of endothelial cells, including Human Microvascular Endothelial Cells (HMEC), Human Pulmonary Artery Endothelial Cells (HPAEC), and HUVEC. We observed the morphology and alterations in marker endothelial molecules of these endothelial cells in both two-dimensional (2D) and three-dimensional (3D) cultures, and compared their growth rate and viability. Immunofluorescence (IF) analysis detected the expression of vascular endothelial marker protein CD31 in the HMEC and HPAEC (Figure 3A). We also observed that, unlike 2D endothelial cell culture, 3D-cultured endothelial cells on Matrigel extended and branched from numerous endothelial cell node centers, forming interconnected vascular networks. This enhanced endothelial cell viability and increased the expression of endothelial markers. Specifically, in 2D culture, HUVEC exhibited minimal CD31 expression; however, after forming a 3D structure with mutual contact on Matrigel, the expression of CD31 on the cell surface became evident (Figure 3B). Although HMEC and HPAEC exhibited strong CD31 expression, their high proliferation rates were found to compromise islet tissue viability during coculture. We thus selected HUVEC for its lower proliferation rate in coculture with islet organoids.

Initially, pancreatic islet cell clusters were seeded onto Matrigel stabilized for 12 h, and HUVEC were added at a 1:1 ratio (islets: endothelial cells). Following 24 h of culture, it was observed that the vascular endothelial cells encapsulated the pancreatic islet cell clusters and formed a network structure (Figure 3C). CD31 immunostaining confirmed HUVEC viability, with network-associated cells showing optimal CD31 expression (Figure 3C). Subsequently, on the day 5 of co-culture, we examined the insulin secretion capacity of the vascularized pancreatic islet organoids. The results showed that the pancreatic islet organoids encapsulated by the vascular network displayed robust insulin secretion ability, indicating high β-cell activity.

### 3.4. Vascularized Islet-like Organoids Exhibit Good Physiological Activity

**Figure 4:**
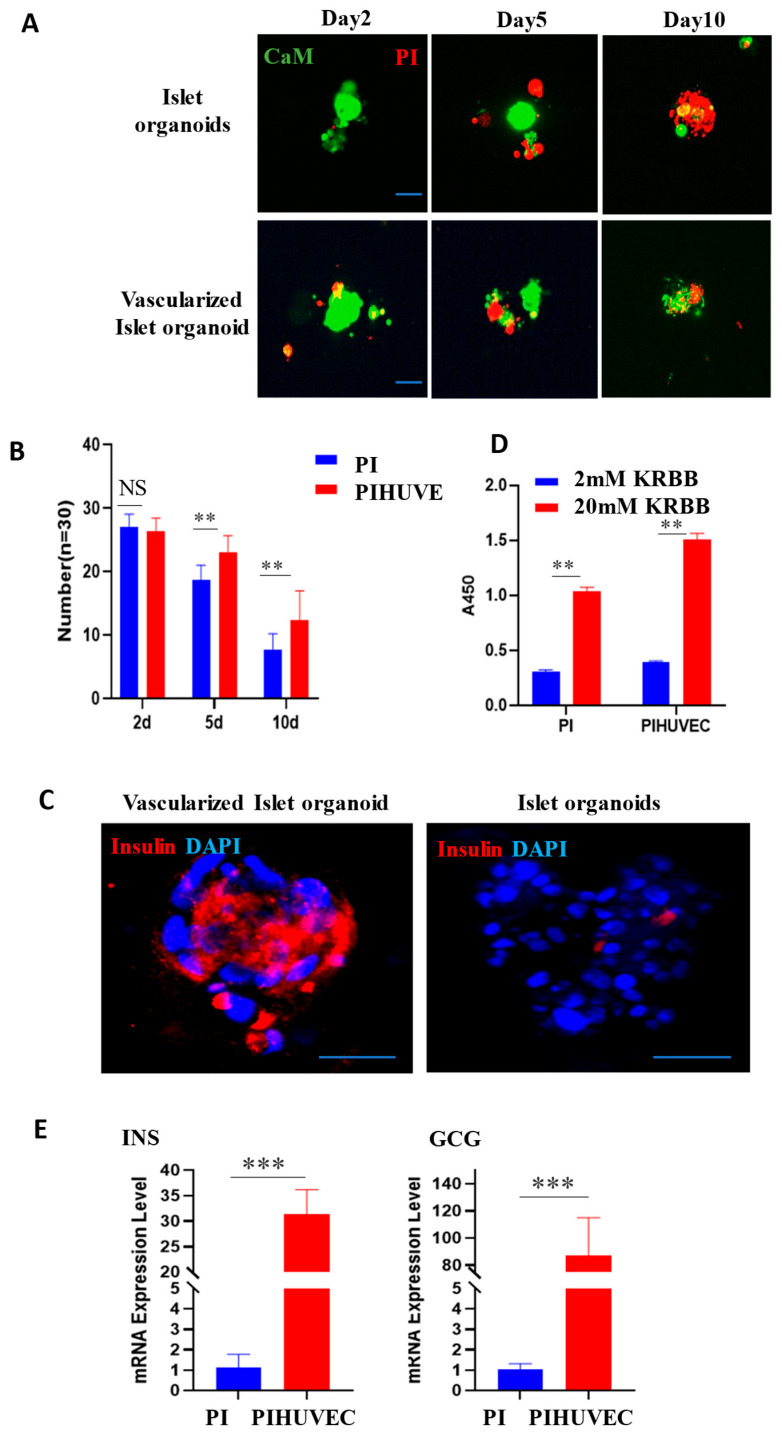
Endothelial cells contribute to survival and function of pancreatic islet organoids. (**A**) After co-culturing of pancreatic islets with endothelial cells, cell viability staining was performed using CaM and PI on the 2nd, 5th, and 10th days. Scale bar, 20 μm. (**B**) Statistical analysis of organoids. The number of surviving islets was counted on the 2nd, 5th, and 10th days of individual culture and co-culture, respectively. ** *p* < 0.01. (**C**) On the 5th day after the co-culturing of pancreatic islets and endothelial cells, the insulin secretion capacity and the expression of vascular endothelial marker molecule CD31 were detected. Scale bar, 20 μm. (**D**) The relative insulin secretion level of islet-like organoids cultured independently and those co-cultured with HUVEC under high-glucose stimulation. (**E**) The mRNA levels of INS and GCG in islets-like organoids cultured independently and those co-cultured with HUVEC on the 5th day. *** *p* < 0.001.

Following the separate culturing and identification of pancreatic islets and vascular endothelial cells, the pancreatic islet cell clusters were co-cultured with HUVEC. We further conducted statistical analyses on the cell survival rate and insulin secretion capacity of both monocultured islet organoids and vascularized islet organoids. For viability assessment, we applied Calcein-AM and propidium iodide (PI) dual staining on days 2, 5, and 10 of culture, quantifying viable cell counts. Results revealed significantly higher survival rates in vascularized organoids over time, with the most pronounced difference on day 10 (Figure 4A,B), demonstrating that vascularization prolongs islet viability in vitro. Subsequently, we conducted glucose-stimulated insulin secretion (GSIS) assays on day 5. Vascularized organoids exhibited stronger insulin secretion than monocultured controls under both low and high glucose conditions, particularly enhanced under high glucose (Figure 4C). Furthermore, qPCR analysis on day 5 showed upregulated mRNA levels of insulin and glucagon in vascularized organoids (Figure 4D,E). In summary, HUVEC coculture significantly enhances in vitro islet survival, upregulates key islet hormone, and improves glucose-responsive function.

## 4. Discussion

Given the crucial role of vascular structure in islet organoids, it is essential to explore a method for constructing vascular networks within these organoids and establish a simulated islet organoid system that incorporates a complete vascular system. At this stage, emphasis should be placed on guiding vascular endothelial cells and other relevant vascular cell types during the process, enabling them to form a three-dimensional islet organoid in conjunction with the islets [19,20,21,22].

This study established a vascularized islet organoid model through optimized isolation and coculture protocols. Human pancreatic tissue was processed to isolate functional islet clusters, which were subsequently cultured in 3D Matrigel systems supplemented with growth factors to generate viable organoids. Comparative screening identified HUVEC as the optimal endothelial partner, leveraging Matrigel to drive tubular network formation. Critically, we demonstrated that endothelial cells significantly enhance islet survival and function, potentially mediated by paracrine crosstalk, though the precise mechanisms remain elusive. The specific mechanisms by which vascularization influences the fate and function of pancreatic organoids still require further elucidation. The precise mechanism by which HUVECs enhance islet survival and function—potentially mediated through paracrine interactions—remains unclear, necessitating further investigation to decipher the molecular dialog governing this synergy.

This work provides a robust platform for studying islet-endothelial interactions, yet further investigation is needed to decode the molecular dialog governing this synergy. Through systematic refinement of islet isolation protocols, we established a reliable vascularized islet organoid culture system. During the initial phase of methodology establishment, vascularized islet organoids exhibited functional impairments, including flotation and death by day 5. Through literature-guided optimization of the islet: HUVEC ratio (3:1) [23,24], these adverse effects were resolved, resulting in >42.30% viability retention and stabilized vascular networks. In initial experiments, we employed a 3:1 (HUVEC:islet) co-culture ratio; however, this configuration yielded suboptimal results. Specifically, the rapid proliferation and metabolic activity of HUVECs disrupted the structural integrity of the islet-endothelial coculture, leading to premature system collapse and compromised islet viability. Subsequent optimization revealed that a balanced 1:1 (HUVEC:islet) ratio significantly improved stability, prompting its adoption as the standardized co-culture strategy for all subsequent analyses.

Furthermore, we discovered that the concentration of VEGF in the culture system exerted a notable influence on the microvascular network. Concentrations below 10 ng/mL induced the disintegration of the microvascular network within vascularized organoids, which was more prominent in islet organoids lacking pericyte support. Future studies could explore angiogenin supplementation to enhance endothelial function, given its established role in promoting endothelial invasiveness and tubulogenesis. Additionally, we observed indications of the differentiation of certain exocrine pancreatic glands into endocrine cells and endothelial cells, consistent with literature reports [25,26,27]. This phenomenon may be attributed to NGN3-positive progenitor cells residing in pancreatic ducts, which undergo differentiation into β cells under hyperglycemic stress and concurrent pancreatic β-cell damage [28,29]. Although such transdifferentiation occurs in diabetic patients, the quantity of functionally mature β cells generated is insufficient to reverse hyperglycemia [30]. Consequently, transplantation of vascularized islet organoids with enhanced insulin secretion capacity remains essential for sustainable diabetes management.

As one of the pivotal factors in the pancreatic islet ecological environment, the intra-islet microvasculature plays a crucial role in its development and orchestrates key physiological and pathological events in pancreatic endocrine homeostasis [31]. In the early stages of pancreatic islet development, upon being stimulated by the Vascular Endothelial Growth Factor (VEGF) secreted by the pancreatic epithelium, it is involved in regulating the development as well as the alterations in hormone production and function within the body [32,33]. Furthermore, in vitro co-culture systems demonstrate that endothelial cells spontaneously aggregate and form tight junctions Studies indicate elevated insulin and PDX1 expression in pancreatic tissues with intact endothelial interactions, suggesting their capacity to support islet cell maturation [34]. Recent research has achieved functional insulin-producing cell differentiation from progenitor cells using basement membrane matrix-based culture [35]. However, prolonged application of such approaches risks significant β-cell loss due to inadequate oxygen or nutrient supply. Therefore, developing vascularized islet organoids is essential to address this limitation. Recent research by Wang et al. has demonstrated the successful utilization of a defined medium to drive the differentiation of progenitor cells into functional β-cells within a Matrigel-based culture system to address this limitation, engineered vascularization emerges as a promising strategy [23,24].

Our islet organoid generation platform is uniquely characterized by its innovative cell source approach. Instead of employing the directed differentiation of pluripotent stem cells, we develop three-dimensional islet-like microtissues through the direct culture of freshly isolated primary islets. This methodology preserves the intrinsic functionality and maturity of adult β-cells and other endocrine cell populations. The resulting architectural organization establishes a robust, physiologically relevant ex vivo system that is particularly well-suited for investigating fundamental aspects of islet biology—including the poorly understood presence and functional significance of resident progenitor cells.

## Figures and Tables

**Table 1:** Culture medium of pancreatic islet cells.

Component	Concentration	Function
DMEM/F12	500 mL	Nutrients necessary for life activities
B27	1×	Maintains long-term in vitro culture of cells
Penicillin and Streptomycin	1×	Antibiotics to prevent contamination
HEPES	10 mM	Buffering agent
Glutamine	2 mM	Provides energy to support the synthesis of proteins and nucleic acids
heparin	2.5 µg/mL	Viscopolysaccharides with anticoagulant properties
FGF2	10 ng/mL	Affects cell proliferation and tissue neovascularization
EGF	50 ng/mL	Mitogenic factor
IGF-1	100 ng/mL	Promotes growth activity and activates the AKT signaling pathway
VEGF	5 ng/mL	Relates to fetal and adult angiogenesis
ITS	100×	Promotes cell growth and regulates nutrient intake

## Data Availability

All data generated or analyzed during this study are included in this published article.

## Abbreviations

The following abbreviations are used in this manuscript:

HUVEC	Human umbilical vein endothelial cells
VEGF	Vascular Endothelial Growth Factor
FGF	Fibroblast Growth Factor
EGF	Epidermal Growth Factor
iPSCs	Induced Pluripotent Stem Cells
ESC	Embryonic Stem Cells
INS	Insulin
GCG	Glucagon
